# Protein model accuracy estimation based on local structure quality assessment using 3D convolutional neural network

**DOI:** 10.1371/journal.pone.0221347

**Published:** 2019-09-05

**Authors:** Rin Sato, Takashi Ishida

**Affiliations:** Department of Computer Science, School of Computing, Tokyo Institute of Technology, Ookayama, Meguro-ku, Tokyo, Japan; University of Michigan, UNITED STATES

## Abstract

In protein tertiary structure prediction, model quality assessment programs (MQAPs) are often used to select the final structural models from a pool of candidate models generated by multiple templates and prediction methods. The 3-dimensional convolutional neural network (3DCNN) is an expansion of the 2DCNN and has been applied in several fields, including object recognition. The 3DCNN is also used for MQA tasks, but the performance is low due to several technical limitations related to protein tertiary structures, such as orientation alignment. We proposed a novel single-model MQA method based on local structure quality evaluation using a deep neural network containing 3DCNN layers. The proposed method first assesses the quality of local structures for each residue and then evaluates the quality of whole structures by integrating estimated local qualities. We analyzed the model using the CASP11, CASP12, and 3D-Robot datasets and compared the performance of the model with that of the previous 3DCNN method based on whole protein structures. The proposed method showed a significant improvement compared to the previous 3DCNN method for multiple evaluation measures. We also compared the proposed method to other state-of-the-art methods. Our method showed better performance than the previous 3DCNN-based method and comparable accuracy as the current best single-model methods; particularly, in CASP11 stage2, our method showed a Pearson coefficient of 0.486, which was better than those of the best single-model methods (0.366–0.405). A standalone version of the proposed method and data files are available at https://github.com/ishidalab-titech/3DCNN_MQA.

## Introduction

The three-dimensional (3D) structure of a protein is related to its function and is important for life science applications such as drug discovery; however, experimentally determining three-dimensional protein structures is costly and time-consuming. Thus, many computational methods for predicting protein 3D structures from amino acid sequences have been developed [1–4]. Current prediction schemes often output multiple structure models because homology searching typically detects multiple template structures, and multiple candidates are generated for each alignment through energy minimization of the structures. Additionally, multiple prediction methods may be used because no single method shows the best performance for each protein. Thus, a near-native model must be selected from a pool of predicted models. Various methods have been developed to evaluate protein structure models to select the best model [5]. These methods are generally referred to as model quality assessment programs (MQAPs).

MQAPs can be divided into two types: single-model methods [6–12] and consensus methods [13–15]. A single-model method can be applied to an individual protein model independently. In contrast, consensus methods require multiple models because they evaluate a protein model while referring to other predicted models. In Critical Assessment of Techniques for protein Structure Prediction (CASP) [16] experiments, consensus methods show better performance [14]. Consensus methods in CASP can use hundreds of high-quality models, but it is difficult to obtain such a large dataset in practical cases. Thus, the availability of a single-model method is greater than that of consensus methods. Moreover, single-model methods often show better performance when the predicted models contain many low-quality models [6].

Many single-model methods have been proposed. Most existing single-model methods utilize high-level features for assessment. For example, SVMQA used 8 potential energy-based features and 11 consistency-based features between the predicted and actual values of the model [6]. DeepQA used 6 potential-based features and 3 single-model method features [11]. ProQ2 used consistency-based features and chemical property features as well as evolutionary information [8]. These methods use high-level features, including evolutionary information and predicted structure property, among others, to achieve more accurate assessment. However, such high-level features are sometimes unavailable, particularly for completely new proteins. Thus, methods assessing model quality only based on protein tertiary structures are required.

Recently, deep learning methods have greatly contributed to several fields, such as speech recognition [17] and image recognition [18]. These deep learning methods often use low-level features such as RGB values of each pixel in images as the input and show better accuracies than non-deep learning methods with high-level features. In such studies, convolutional neural networks (CNNs) are often used rather than general neural networks. Two-dimensional CNNs (2DCNNs) have been effectively applied in the image recognition field [18,19]. Three-dimensional convolutional neural networks (3DCNNs) have been proposed and showed higher accuracy in object recognition [20]. 3DCNN is also applied in bioinformatics applications, such as protein binding-site detection [21] and for predicting protein-ligand absolute binding affinity [22].

Derevyanko *et al*. used 3DCNN for MQA applications [23]. Their method defined a single 120-Å bounding box surrounding each protein structure, followed by grid featurization of the bounding box and 3DCNN training. However, the large single bounding box-based method has two limitations: (1) the bounding box size problem and (2) the orientation of the box. For the first limitation, it is difficult to determine the appropriate bounding box size because protein structures are not uniform in size. If the size of bounding box is too large, the contents of the box become too sparse. If bounding box size is too small for a target protein, the whole protein structure cannot be evaluated. The second limitation involves difficulty in aligning the orientations of proteins, as protein structures have no specific orientation. Thus, the authors rotated and translated the structure randomly 90 times and averaged the score. However, the number of rotations was too small to account for the total number of possible rotations and translations (for example, 5,400 patterns exist if the sampling step uses a 15° rotation). Additionally, even if enough rotations and translations were applied, the redundant dataset generated may cause over-fitting during the training processes. To solve this problem, Pagès *et al*. proposed a residue-wise scoring function (Ornate) that uses 3D density maps as input which corresponds to each residue and its neighboring residues with the backbone topology of the residue [24]. This approach succeeded to avoid the problem of ambiguous orientations of the initial models. However, the method proposed by Pagès *et al*. uses complex inputs and network topology for the neural network, and thus the performance of the method was lower than that of state-of-the-art single MQA methods.

In this study, we developed a novel MQA method based on a residue-wise assessment method for evaluating the local structure of each residue using 3DCNN. The proposed method sets a small bounding box for each residue, and thus the orientations of the boxes can be determined using main chain coordinates. We used simpler atom categories and network topologies that could be easily trained. We applied the proposed method and existing methods to the benchmarking datasets CASP11, CASP12, and 3DRobot [25]. The proposed method showed significantly better accuracy than the 3DCNN-based method developed by Derevyanko. Additionally, the proposed method exhibited the best accuracy compared to other state-of-the-art single-model methods.

## Materials and methods

**Fig 1:**
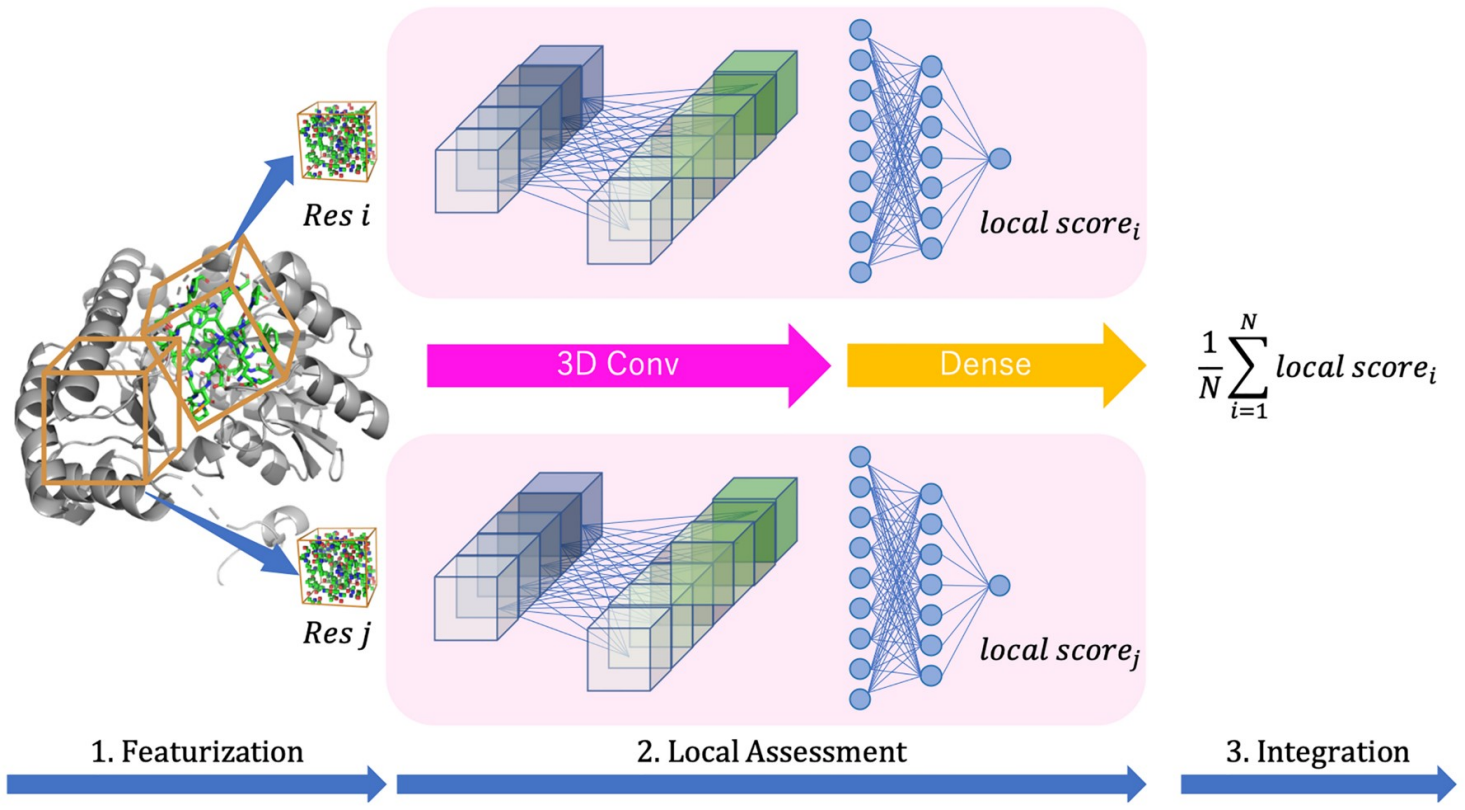
Workflow of proposed method. 1. Local structure was extracted by 3D grid bounding box for each residue. 2. Local structure quality was evaluated using 3D convolutional neural network. 3. Integration residue-wise local score into whole structure score.

In contrast to the previous method developed by Derevyanko *et al*. [23] which uses a single large bounding box for the whole protein structure, our proposed method is based on residue-wise 3DCNN, which evaluates the local structure of a residue using 3DCNN. This method assumes that the local structure quality implies global quality. The workflow of the proposed method is shown in Fig 1. The procedure is separated into three steps: (1) residue-wise low-level featurization, (2) 3DCNN-based local structure assessment, and (3) integration of residue-wise local results.

### Residue-wise low-level featurization

**Fig 2:**
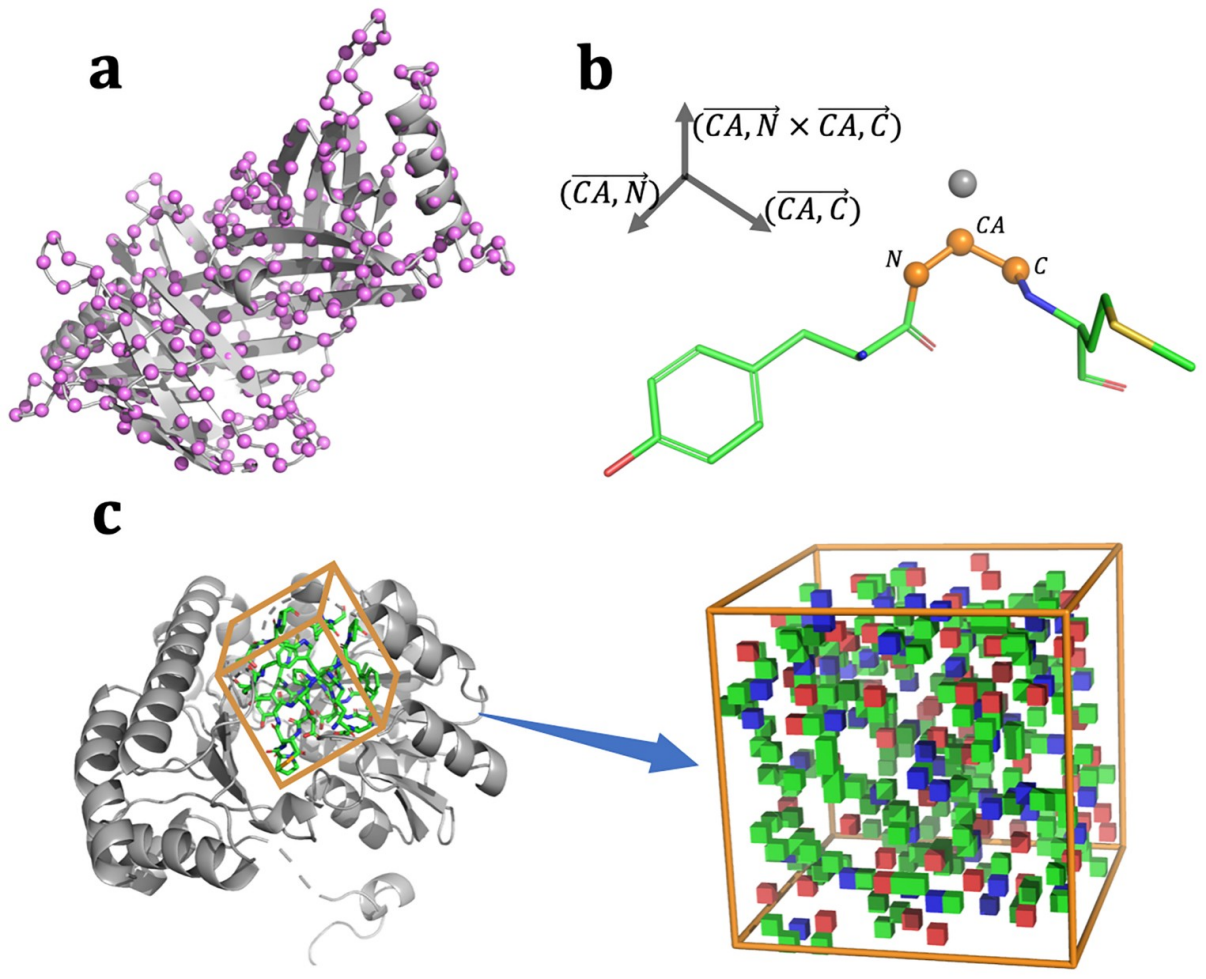
Featurization of local structure. (a) 3D grid bounding box was set for each C-alpha atom (CA) of a residue. One side size of the box was 28 Å and the box was divided into 1-Å voxels. (b) The orthonormal basis of the bounding box was calculated from C-CA vector and N-CA vector and cross product of C-CA and N-CA. (c) Atoms featured within a voxel were labeled into 14 categories as shown in Table 1. Each category feature was assigned into an independent channel of the CNN. In the figure, each voxel is colored as C, N, O, and S.

To extract input data for a neural network from a protein structure, we first set a 3D grid bounding box centered by the C-alpha atom (CA) of a residue. One side of the box was 28 Å and the box was divided into 1-Å voxels. To determine the orientation of the box, the orthonormal basis calculated from the C-CA vector and N-CA vector and cross-product of the C-CA and N-CA vector was used as the axis of the bounding box according to similar definitions used in a related study [26]. Fig 2(b) shows how the orientation was determined. Atoms within a voxel were checked and the features were assigned to the voxel. Atom features were placed into 14 categories based on the atom type as shown in Table 1. The 11 categories were used according to a previous study of previous 3DCNN study based on whole protein structures [23]. We added 3 categories (CA atom, backbone chain atom, any atom). Each category feature was assigned to an independent channel of a neural network (Fig 2(c)).

**Table 1:** Atom feature 14 categories.

Type	Description	Atoms
1	Sulfur/selenium	CYS:SG, MET:SD, MSE:SE
2	Nitrogen (amide)	ASN:ND2, GLN:NE2, backbone N (including N-terminal)
3	Nitrogen (aromatic)	HIS:ND1/NE1, TRP:NE1
4	Nitrogen (guanidinium)	ARG:NE/NH*
5	Nitrogen (ammonium)	LYS:NZ
6	Oxygen (carbonyl)	ASN:OD1, GLN:OE1, backbone O (except C-terminal)
7	Oxygen (hydroxyl)	SER:OG, THR:OG1, TYR:OH
8	Oxygen (carboxyl)	ASP:OD*, GLU:OE*, C-terminal O, C-terminal OXT
9	Carbon (sp2)	ARG:CZ, ASN:CG, ASP:CG, GLN:CD, GLU:CD, backbone C
10	Carbon (aromatic)	HIS:CG/CD2/CE1,PHE:CG/CD*/CE*/CZ, TRP:CG/CD*/CE*/CZ*/CH2, TYR:CG/CD*/CE*/CZ
11	Carbon (sp3)	ALA:CB, ARG:CB/CG/CD, ASN:CB, ASP:CB, CYS:CB, GLN:CB/CG, GLU:CB/CG, HIS:CB, ILE:CB/CG*/CD1, LEU:CB/CG/CD*, LYS:CB/CG/CD/CE, MET:CB/CG/CE, MSE:CB/CG/CE, PHE:CB, PRO:CB/CG/CD, SER:CB, THR:CB/CG2, TRP:CB, TYR:CB, VAL:CB/CG*, backbone CA
12	Occupancy	*:*
13	Backbone	*:N,*:CA,*:C
14	CA	*:CA

1–11 atom types were cited from Derevyanko et al. [23]. We also added 3 classes (CA atom, backbone chain atom, all atoms).

### 3DCNN-based local structure assessment

In this step, we evaluated the local structure of a residue based on the voxel information generated in the previous step by supervised machine learning. To train the supervised machine learning model, a label indicating the local structure quality of each residue was required. We used lDDT as a label to describe the local structure quality [27]. To overcome the binary classification problem, the label was defined using the following formula:

$$local\;label=\left\{ \begin{array}{cc} 1 & if\;lDDT>0.5\;\\ 0 & (otherwise) \end{array}\right.$$

To predict local structure quality, we used a deep neural network including 3DCNN layers. A 3DCNN is an expansion of a 2DCNN, which is often used in image recognition. 3DCNN is used for object recognition [20] and can effectively extract features from 3D structured data, as conducted in feature learning of 2DCNN for image recognition. S1 Fig shows the neural network architecture. We designed the architecture based on previous 3DCNN research [26]. The last 3DCNN layer was connected to the global average pooling layer [28]. After each layer, PReLU [29] was used as the activation function other than the output layer. The batch normalization layer [30] was added before each activation function. The prediction problem is a binary classification, and thus sigmoid cross entropy was used as a loss function.

### Integration of local results

A neural network for local structure assessment returns an estimated quality value for each residue. Thus, we integrated the local scores into a global score to evaluate the quality of a whole protein structure. It is difficult to use machine-learning methods to integrate these scores because the number of local scores is not fixed. Thus, the global score was simply calculated as the mean value of the local scores.

### Dataset

To train the local structure assessment models, native structures and non-native decoy structures were collected from the targets and prediction results of CASP experiments [16]. We used the CASP 7–10 datasets obtained from the CASP homepage (http://predictioncenter.org/download_area/) for training. Decoy structures were 10% randomly sampled for each target protein in the training datasets (26.6 models per protein were used). In total, the training dataset contained 11,582 protein structure models for 435 proteins. The training dataset included 968,869 positive data and 958,780 negative data. We used scwrl4 [31] to optimize side-chain conformations, as used in a previous 3DCNN study based on whole protein structures.

For the test datasets, we used the CASP11, 12 datasets and 3DRobot decoy sets [25], which were used in a previous study by Derevyanko et al. [23]. The test datasets from CASP include stage1 and 2 decoys; stage1 uses up to 20 selected predictions spanning the whole range of model accuracy and stage2 uses the best 150 server predictions according to the ranking from the DAVIS-EMAconsensus method [32]. Additionally, Targets T0797, T0798, and T0825 in CASP11 were removed from the benchmark because they were released for multimeric prediction. Similarly, we used scwrl4 for the test datasets. Table 2 shows additional details of the datasets. GDT_TS in the Results section was calculated by using TMscore [33]. When using TMscore, a target structure and model structure must be specified and a different value is returned if the model structure is considered as a target structure and target structure is considered as a model structure. In the Results section, GDT_TS was calculated using a target structure and model structure in an inverted manner according to Derevyanko *et al*. [23]. The results for non-inversed GDT_TS are shown in S1 Table.

**Table 2:** Decoy set detail used for comparison to previous 3DCNN method based on whole protein structures.

Decoy set	Number of decoys per target	Number of targets
CASP11 stage1	20.0	81
CASP11 stage2	148.2	80
CASP12	172.1	40
3DRobot	300.0	200

### Evaluation

We used the correlation between the predicted quality scores and GDT_TS values of models as evaluation measures. We used Pearson’s correlation coefficient and Spearman’s correlation as the test datasets. A test dataset contained many target proteins, and the correlations were calculated for each target. Thus, we used the average of these values. We also evaluated the near-native selection performance of the method using two measures. We determined the difference value between the GDT_TS of a selected model by each assessment method and that of the best GDT_TS model (GDT_TS loss). We also used the score-based rank of the best GDT_TS model (best model rank).

## Results

### Neural network training for local structure assessment and performance evaluation

**Fig 3:**
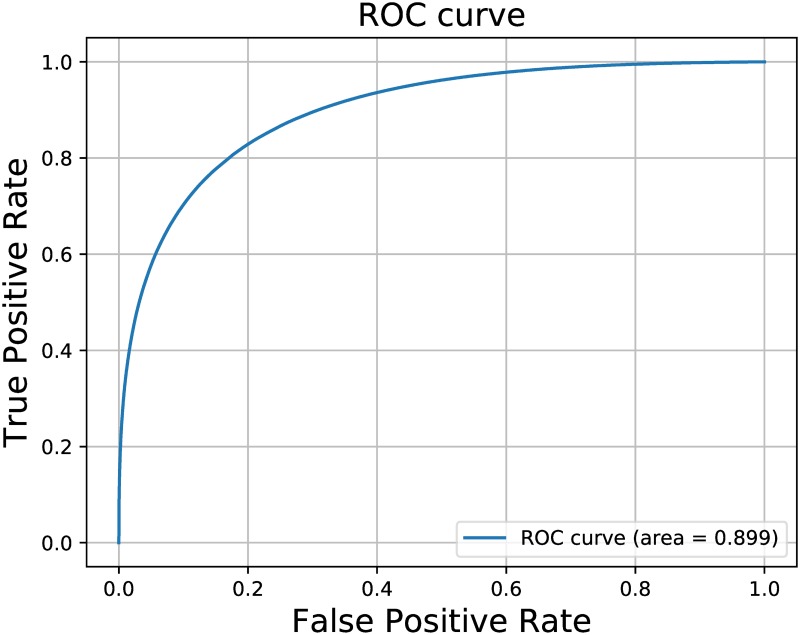
ROC curve of best epoch model. ROC curve of best validation loss epoch model.

We first trained a deep neural network including 3DCNN layers to assess the local structure quality of a residue. Thus, this analysis is based on the binary classification problem for each residue. We split the training set into a “neural network training set” and “validation set” with a split rate of 80% in target protein level. The validation dataset was used to determine the hyperparameters of a network. To evaluate the accuracy of the networks, we constructed a receiver operator characteristics (ROC) curve [34]. The area under the ROC curve (ROC_AUC) of the validation set was used to determine the stopping epoch in the training. In this study, one epoch was determined as the end of once training with whole the training data. We used SMORMS3 at a learning rate of 0.001 [35], which is the default value, for optimization. The loss and AUC value during training are shown in S2 Fig. The loss and MCC value using prediction threshold 0.5 are also shown in S3 Fig. Fig 3 shows the ROC curve of the best epoch model, which showed an ROC_AUC of 0.906. This indicates that the trained model can be applied to assess local structure quality.

### Model quality assessment performance evaluation

The previous section described that the proposed 3DCNN-based model achieved high accuracy for local structure quality assessment. However, to estimate the performance of the proposed method for assessing the quality of a whole protein structure model, other evaluation experiments should be performed.

To determine the performance of the proposed method, we performed two performance evaluations. The first involved comparison to a 3DCNN method based on whole protein structures [23]. We also performed another performance evaluation to compare the performance of the proposed method with state-of-the-art single MAQ methods because the method described by Derevyanko et al. does not currently give the best results [23].

### Performance comparison with a previous 3DCNN method based on whole protein structures

We evaluated the model performance using the CASP11, CASP12, and 3DRobot decoy sets and compared the results with those from the 3DCNN method developed by Dereveyanko et al. [23]. We compared the performance of the proposed method with the values obtained from the previous 3DCNN method based on whole protein structures in the article [23]. Table 3 shows the results of the evaluation tests. The values of the previous 3DCNN method were obtained from the article. The results showed that proposed method achieved better performance than the previous 3DCNN method for all measures. To confirm the significance of the improvement, we performed a Wilcoxon signed-rank test. The values in parenthesis under the values obtained using the proposed method are the p-values determined in statistical analysis. The score of the previous method for CASP12 was not available, and thus we did not perform the test in this case. The improvement was significant for all datasets. The results indicate that the proposed method is superior to the 3DCNN method based on whole protein structures in the MQA task.

**Table 3:** Comparison with previous 3D-CNN method.

Dataset	Method	Pearson	Spearman	GDT_TS loss	Best model rank
CASP11 stage1	Proposed	**0.661**	**0.531**	**5.739**	**3.136**
	Derevyanko+2018	0.535 **(1.43E-08)**	0.425 **(2.37E-06)**	6.396	3.691
CASP11 stage2	Proposed	**0.500**	**0.471**	**5.000**	**22.425**
	Derevyanko+2018	0.421 **(1.42E-05)**	0.409 **(1.03E-03)**	6.449	27.563
CASP12	Proposed	**0.652**	**0.614**	**14.557**	44.200
	Derevyanko+2018	0.607 (NA)	0.521 (NA)	**14.6**	NA
3DRobot	Proposed	**0.931**	**0.882**	**1.708**	**4.290**
	Derevyanko+2018	0.856 **(4.62E-53)**	0.839 **(1.26E-22)**	9.627	18.610

The first and second columns represent the dataset name and method name. The third and fourth columns, respectively, show the average Pearson’s correlation coefficient (Pearson) and average Spearman’s correlation (Spearman) between the actual ranking and predicted ranking. The fifth and sixth column show the average GDT_TS loss and best model rank. Values in parenthesis in the columns 3–4 show the p-value (Wilcoxon signed-rank test) for the differences in Pearson and Spearman results, respectively, between the proposed method and previous method (Derevyanko+2018). A p-value <0.05 indicates that the difference was significant. Values with high accuracy and p-values <0.05 are shown in bold.

### Comparison to state-of-the-art methods

We also performed another performance evaluation to compare the performance of the proposed method with the best-performing single-model QA methods according to CASP11,12 assessment: SVMQA [6], ProQ2 [8], ProQ2-refine [8], ProQ3 [12], RFMQA [7], VoroMQA [9], MULTICOM-CLUSTER [10], and MULTICOM-NOVEL [10]. Additionally, we also conducted comparison with Ornate [24], which is a recent single-model QA method that uses residue-wise 3DCNN. For this evaluation, we used the dataset used in CASP official assessments. This dataset was slightly different from the dataset used in the previous section and included GDT_TS labels. The details of the dataset are shown in S2 Table. The CASP11 results of ProQ2, ProQ2-refine, RFMQA, VoroMQA, MULTICOM-CLUSTER, and MULTICOM-NOVEL were extracted through blind prediction of CASP11. The CASP12 results of ProQ2, SVMQA, ProQ3, VoroMQA, and MULTICOM-CLUSTER were similarly extracted through blind prediction of CASP12. Only the results of Ornate were extracted from the previous article [24]. CASP11,12 stage2 results are shown in Tables 4 and 5 and CASP11,12 stage 1 results are shown in S3 and S4 Tables. The score of Ornate for each target was not available, and thus we did not perform statistical analysis in this case. The proposed method achieved better or comparable accuracy, particularly in CASP11 stage2, and the proposed method outperformed the other methods evaluated.

**Table 4:** Comparison with single-model methods in CASP11 stage2.

Method	Pearson	Spearman	GDT_TS loss	Best model rank
Proposed	**0.486**	**0.452**	**4.945**	**26.977**
VoroMQA	0.413 **(0.0009)**	0.394 **(0.0080)**	7.307	27.25
MULTICOM-CLUSTER	0.405 **(0.0001)**	0.397 **(0.0068)**	7.058	31.83
MULTICOM-NOVEL	0.390 **(5.73E-05)**	0.389 **(0.0073)**	6.888	32.375
RFMQA	0.369 **(1.52E-06)**	0.351 **(2.64E-05)**	7.021	31.621
ProQ2	0.368 **(4.89E-07)**	0.363 **(9.51E-05)**	6.34	35.705
ProQ2-refine	0.366 **(3.30E-07)**	0.373 **(0.0003)**	6.754	34.67
Ornate	0.39 (NA)	0.37 (NA)	5.5	NA

The legend is the same as that for the columns 2–6 in Table 3.

**Table 5:** Comparison to single-model methods in CASP12 stage2.

Method	Pearson	Spearman	GDT_TS loss	Best model rank
Proposed	**0.665**	**0.594**	6.159	**16.563**
ProQ3	0.639 **(0.0124)**	0.590 (0.8081)	5.633	20.343
SVMQA	0.631 (0.2575)	0.587 (0.5408)	**5.261**	20.743
VoroMQA	0.593 **(4.41E-06)**	0.544 **(0.0030)**	7.789	19.914
ProQ2	0.591 **(2.97E-05)**	0.556 **(0.0176)**	6.823	20.843
MULTICOM-CLUSTER	0.577 **(5.51E-06)**	0.540 **(0.0233)**	7.678	24.543
Ornate	0.49 (NA)	0.46 (NA)	7.200	NA

The legend is the same as that for columns 2–6 in Table 3.

## Discussion

### Influences of homologues between training and test datasets

We used similar training and test sets as used in previous studies. However, the test dataset included several homologues proteins to those in the training dataset. To evaluate the influence of homologues, we removed proteins with sequence similarity to those in the training set from the test set. We used NCBI BLASTP [36] and an e-value threshold of >1e-4 to identify the homologues. There were 8 homologues in the CASP11 dataset and 6 homologues in the CASP12 dataset. The detailed information is shown in S5 Table. S6–S9 Tables show the accuracy of model quality assessment without homology proteins for each test dataset. The accuracies of the proposed method for the non-homologue dataset were nearly the same as those in the Results section (for instance, Pearson’s correlations for CASP11 stage2 dataset were 0.486 and 0.483, respectively). Additionally, the improvement compared to other state-of-the-art methods did not change. The information of homologues proteins is often useful for this application. However, we disintegrate the problem to the residue-level, and thus the influence was not critical.

### Evaluation on non-CASP datasets

In the Results section, we mainly used datasets from the CASP experiments. The datasets were major in this field [23, 24]. However CASP datasets were constructed for a competition so that the targets were not systematically selected. Thus, they are not perfectly non-redundant and do not cover whole protein structure space. Thus, we also evaluated non-CASP datasets: 3DRobot decoy set [25] and I-TASSER decoy set II [37]. The native structures were removed from all datasets. Protein sidechain structures were optimized by using Scwrl4 and the ground truth label was TMscore calculated by using TMscore software. SVMQA [6], RWplus [37], GOAP [38], and OPUS-PSP [39] were compared to the proposed method. Accuracies were extracted from the article [6]. The results are shown in S10 and S11 Tables. For the 3DRobot dataset, the proposed method showed comparable accuracies to SVMQA and outperformed the other methods. For the I-TASSER dataset, the proposed method also showed better accuracy than the other methods except for SVMQA but the accuracy of SVMQA was better in all measures. Our data did not reveal why SVMQA showed better accuracy with the I-TASSER dataset compared to that for the other test sets. In this comparison, only SVMQA used high-level information such as evolutionally information. Thus, such information may be effective for the I-TASSER dataset.

### Performance of local structure assessment

In protein structure model quality assessment, local structure assessment, which evaluates the quality of a structure model in residue-level, is also important because a user can recognize which substructure needs to be improved. Although proposed method is for assessing the quality of a global structure, it outputs a score for each residue in the evaluation process. Thus, we also evaluated the accuracy of local structure assessment of proposed method based on per-residue error estimation. To evaluate the performance of local structure assessment, we used CASP12 stage2 dataset. In the dataset, a model structure and a native structure were superimposed by local-global alignment (LGA) [40] and the distance between a model structure and a native structure for a residue can be calculated. According to CASP assessment [16], we evaluated proposed method using two metrics. One is Pearson correlation coefficient between distances and predicted scores. The other is the ROC-AUC by considering the problem as binary classification. If a distance is smaller than 3.8Å, the prediction of a residue is considered as correct. S12 Table shows the result of local assessment evaluation. We compared the accuracy of proposed method with other single model assessment methods. We used only methods which can predict residue-wise quality. Proposed method showed comparable performance with the other methods in AUC. In contrast, the performance by Pearson correlation coefficient was the worst. This result seems to be reasonable because proposed method was trained as a binary classification, and thus it is difficult to estimate the quality of a local structure quantitatively. To improve local structure assessment accuracy by Pearson correlation coefficient, we might change the problem from binary classification to regression. However, the training of a regression model is often more difficult to a classification model, and we considered it caused the decrease of global structure assessment performance.

### Performance difference in core and surface residues

We investigated the local assessment accuracy of proposed method by dividing residues into core residues and surface residues. Residues on the protein surface often have a small number of contacting residues in the bounding box and insufficient information may decrease the accuracy of assessment. The class of a residue was defined by its relative solvent accessibility area (RSA). If the RSA was less than 25%, the residue was categorized into the core. The ROC-AUC was used to determine local assessment accuracy. The CASP11 stage2 datasets and their RSAs were calculated by using FreeSASA [41].

As a result, local assessment accuracy for the core residues (0.918) was superior to that for the surface residues (0.887). These results support the assumption that core residues are more important and indicated that surface residues may decrease assessment performance. Thus, we compared the quality assessment accuracy of the whole model between the proposed method and method only using core residues for assessment (S13 Table). The method only using core residue assessments showed decreased accuracy, indicating that assessment based on surface residues is more difficult but still useful and needed for better assessment. However, improvements can be made by using more sophisticated integration methods rather than the simple mean value of local assessments.

## Conclusion

We proposed a novel model quality assessment method for protein tertiary structure prediction based on machine learning. The method evaluates the local structure quality of each residue using a deep neural network including 3DCNN layers and assesses the quality of the whole structure through integration. Evaluation tests with multiple datasets revealed that the proposed method achieved better accuracy than the previous 3DCNN method, which evaluates whole protein structures within a single large box. Compared to other state-of-the-art single-model methods, the proposed method showed comparable performance. Particularly, for the CASP11 stage2 dataset, the proposed method significantly outperformed the other methods.

Additional studies are needed to extend the training set. In this study, we used a relatively small dataset containing 435 proteins, but the Protein Data Bank contained more than 140,000 protein structures as of 2018 [42]. Thus, accuracy improvement can be achieved by generating more training sets. Additionally, we used a simple average to integrate the local assessment results because the size of the results was not fixed. However, current neural network techniques can deal with such data and may improve the accuracy of the method. Our method does not use high-level features used in other methods. Thus, using high-level features such as evolutionally information may improve the accuracy.

## Supporting information

S1 Table Comparison with previous 3D-CNN method with different labeling. The legend is the same as that for columns 2–6 in Table 3. GDT_TS was calculated using TMscore with the non-invers native and model structure. Derevyanko+2018 result of CASP12 is not available.
(DOCX)

S2 Table Decoy set detail used for comparison to other methods. The first column represents a decoy set name. The second column shows the average of the number of decoys per target protein. The third column shows the number of target proteins in decoy set.
(DOCX)

S3 Table Comparison with single-model methods in CASP11 stage1. The legend is the same as that for Table 4 for the first five columns.
(DOCX)

S4 Table Comparison with single-model methods in CASP12 stage1. The legend is the same as that for in Table 4 for the first five columns.
(DOCX)

S5 Table Detailed information on homologous proteins in the test dataset. The first column represents the test dataset protein ID. The second and third columns, respectively, show the closest protein ID in train dataset and E-value.
(DOCX)

S6 Table Comparison with single-model methods in CASP11 stage1 without homologous proteins. The legend is the same as that for Table 4 for the first five columns.
(DOCX)

S7 Table Comparison with single-model methods in CASP11 stage2 without homologous proteins. The legend is the same as that for Table 4 for the first five columns.
(DOCX)

S8 Table Comparison with single-model methods in CASP12 stage1 without homologous proteins. The legend is the same as that for Table 4 for the first five columns.
(DOCX)

S9 Table Comparison with single-model methods in CASP12 stage2 without homologous proteins. The legend is the same as that for Table 4 for the first five columns.
(DOCX)

S10 Table Comparison with single-model methods in I-TASSER. The first column represents the method name. The second and third columns, respectively, represent the average Pearson’s correlation coefficient (Pearson) and average Spearman’s correlation (Spearman) between the actual ranking and predicted ranking. The fourth column represents the average TMscore loss. Native structures were removed.
(DOCX)

S11 Table Comparison with single-model methods in 3DRobot. The legend is the same as that for S11 Table for the first four columns.
(DOCX)

S12 Table Local assessment performance comparison with other methods in CASP12 stage2. First column represents method name. Second and third columns represent AUC and Pearson value of local assessment.
(DOCX)

S13 Table Comparison with the method using only core residues local assessment in CASP11 stage2. The legend is the same as that for Table 3 for the first six columns.
(DOCX)

S1 Fig Convolutional neural network architecture. The neural network architecture is shown.
(DOCX)

S2 Fig Loss and AUC during training. Loss values and validation AUC are shown.
(DOCX)

S3 Fig Loss and MCC during training. Loss values and validation MCC are shown.
(DOCX)
