# Supplementary material for: Protein model accuracy estimation based on local structure quality assessment using 3D convolutional neural network
Source: PLoS One. 2019 Sep 5;14(9):e0221347. doi: 10.1371/journal.pone.0221347 (PMC6728020; doi:10.1371/journal.pone.0221347)
Supplement: S1 Table — The legend is the same as that for columns 2–6 in Table 3. GDT_TS was calculated using TMscore with the non-invers native and model structure. Derevyanko+2018 result of CASP12 is not available. (DOCX) [file pone.0221347.s001.docx]

S1 Table. Comparison with previous 3D-CNN method with different labeling

The legend is the same as that for columns 2–6 in Table 3. GDT_TS was calculated using TMscore with the non-invers native and model structure. Derevyanko+2018 result of CASP12 is not available.

| Dataset | Method | Pearson | Spearman | Loss | Rank |
| --- | --- | --- | --- | --- | --- |
| CASP11 stage1 | Proposed | **0.655** | **0.518** | **6.643** | **2.914** |
|  | Derevyanko+2018 | 0.528 **(1.13E-08)** | 0.410 **(1.15E-06)** | 7.866 | 3.654 |
| CASP11 stage2 | Proposed | **0.495** | **0.465** | **4.580** | **23.400** |
|  | Derevyanko+2018 | 0.400 **(4.47E-07)** | 0.390 **(1.22E-04)** | 7.293 | 36.450 |
| CASP12 | Proposed | 0.701 | 0.626 | 9.000 | 17.900 |
|  | Derevyanko+2018 | NA | NA | NA | NA |
| 3DRobot | Proposed | **0.931** | **0.882** | **1.686** | **4.205** |
|  | Derevyanko+2018 | 0.852 **(4.51E-53)** | 0.834 **(1.25E-22)** | 9.603 | 18.535 |
