# Supplementary material for: Protein model accuracy estimation based on local structure quality assessment using 3D convolutional neural network
Source: PLoS One. 2019 Sep 5;14(9):e0221347. doi: 10.1371/journal.pone.0221347 (PMC6728020; doi:10.1371/journal.pone.0221347)
Supplement: S2 Table — The first column represents a decoy set name. The second column shows the average of the number of decoys per target protein. The third column shows the number of target proteins in decoy set. (DOCX) [file pone.0221347.s002.docx]

S2 Table. Decoy set detail used for comparison to other methods

The first column represents a decoy set name. The second column shows the average of the number of decoys per target protein. The third column shows the number of target protein in decoy set.

| Decoy set | Decoy number per target | Target number |
| --- | --- | --- |
| CASP11 stage1 | 20.0 | 88 |
| CASP11 stage2 | 150.3 | 88 |
| CASP12 stage1 | 20.0 | 70 |
| CASP12 stage2 | 149.9 | 71 |
