# Supplementary material for: Protein model accuracy estimation based on local structure quality assessment using 3D convolutional neural network
Source: PLoS One. 2019 Sep 5;14(9):e0221347. doi: 10.1371/journal.pone.0221347 (PMC6728020; doi:10.1371/journal.pone.0221347)
Supplement: S3 Table — The legend is the same as that for Table 4 for the first five columns. (DOCX) [file pone.0221347.s003.docx]

S3 Table. Comparison with single-model methods in CASP11 stage1

The legend is the same as that for Table 4 for the first five columns.

| Method | Pearson | Spearman | Loss | Rank |
| --- | --- | --- | --- | --- |
| Proposed | 0.639 | 0.501 | **6.515** | **2.943** |
| ProQ2-refine | **0.654** (0.7613) | **0.544** (0.1245) | 8.555 | 3.398 |
| MULTICOM-CLUSTER | 0.648 (0.7677) | 0.511 (0.9271) | 9.470 | 3.886 |
| ProQ2 | 0.647 (0.9668) | 0.524 (0.4287) | 8.136 | 3.636 |
| MULTICOM-NOVEL | 0.636 (0.5353) | 0.534 (0.3628) | 9.082 | 4.159 |
| RFMQA | 0.609 **(0.0394)** | 0.497 (0.909) | 9.028 | 4.307 |
| VoroMQA | 0.563 **(0.0004)** | 0.444 (0.132) | 10.761 | 4.341 |
| Ornate | 0.47 (NA) | 0.37 (NA) | 7.7 | NA |
