# Supplementary material for: Protein model accuracy estimation based on local structure quality assessment using 3D convolutional neural network
Source: PLoS One. 2019 Sep 5;14(9):e0221347. doi: 10.1371/journal.pone.0221347 (PMC6728020; doi:10.1371/journal.pone.0221347)
Supplement: S4 Table — The legend is the same as that for in Table 4 for the first five columns. (DOCX) [file pone.0221347.s004.docx]

**S4 Table. Comparison with single-model methods in CASP12 stage1**

The legend is the same as that for Table 4 for the first five columns.

| Method | Pearson | Spearman | Loss | Rank |
| --- | --- | --- | --- | --- |
| Proposed | **0.696** | 0.482 | **2.512** | **2.186** |
| MULTICOM-CLUSTER | 0.677 (0.2158) | 0.608 **(5.59E-05)** | 7.044 | 2.614 |
| ProQ3 | 0.661 **(0.0144)** | 0.638 **(2.81E-06)** | 4.065 | 2.42 |
| SVMQA | 0.651 **(0.0041)** | 0.649 **(9.09E-09)** | 3.271 | 2.414 |
| ProQ2 | 0.635 **(0.0035)** | 0.643 **(3.25E-08)** | 7.41 | 2.771 |
| VoroMQA | 0.610 **(6.87E-06)** | 0.551 **(0.0052)** | 7.671 | 2.743 |
| Ornate | 0.57 (NA) | 0.50 (NA) | 11.3 | NA |
