# Supplementary material for: Protein model accuracy estimation based on local structure quality assessment using 3D convolutional neural network
Source: PLoS One. 2019 Sep 5;14(9):e0221347. doi: 10.1371/journal.pone.0221347 (PMC6728020; doi:10.1371/journal.pone.0221347)
Supplement: S5 Table — The first column represents the test dataset protein ID. The second and third columns, respectively, show the closest protein ID in train dataset and E-value. (DOCX) [file pone.0221347.s005.docx]

**S5 Table. Detailed information of homologous protein in test dataset**

The first column represents the test dataset protein ID. The second and third columns, respectively, show the closest protein ID in train dataset and E-value.

| Test dataset ID | Closest train dataset ID | E-value |
| --- | --- | --- |
| T0768 | T0690 | 1.69E-13 |
| T0770 | T0645 | 1.36E-13 |
| T0772 | T0518 | 1.94E-07 |
| T0783 | T0699 | 1.29E-20 |
| T0798 | T0308 | 6.11E-06 |
| T0813 | T0398 | 1.68E-05 |
| T0819 | T0636 | 5.02E-15 |
| T0854 | T0324 | 1.76E-13 |
| T0873 | T0356 | 2.13E-46 |
| T0887 | T0637 | 8.06E-05 |
| T0889 | T0738 | 5.86E-15 |
| T0907 | T0392 | 4.86E-05 |
| T0910 | T0456 | 1.38E-28 |
| T0931 | T0637 | 8.06E-05 |
