# Supplementary material for: Protein model accuracy estimation based on local structure quality assessment using 3D convolutional neural network
Source: PLoS One. 2019 Sep 5;14(9):e0221347. doi: 10.1371/journal.pone.0221347 (PMC6728020; doi:10.1371/journal.pone.0221347)
Supplement: S6 Table — The legend is the same as that for Table 4 for the first five columns. (DOCX) [file pone.0221347.s006.docx]

**S6 Table. Comparison with single-model methods in CASP11 stage1 without homologous proteins**

The legend is the same as that for Table 4 for the first five columns.

| Method | Pearson | Spearman | Loss | Rank |
| --- | --- | --- | --- | --- |
| Proposed | 0.621 | 0.485 | **6.675** | **2.840** |
| ProQ2-refine | **0.637** (0.7902) | **0.542** **(0.0396)** | 8.942 | 3.398 |
| MULTICOM-CLUSTER | 0.635 (0.9906) | 0.511 (0.5358) | 8.765 | 3.886 |
| ProQ2 | 0.630 (0.9381) | 0.523 (0.1672) | 8.204 | 3.636 |
| MULTICOM-NOVEL | 0.622 (0.7328) | 0.539 (0.0951) | 8.422 | 4.159 |
| RFMQA | 0.596 (0.1008) | 0.496 (0.5714) | 8.684 | 4.307 |
| VoroMQA | 0.559 **(0.0026)** | 0.450 (0.4007) | 10.557 | 4.341 |
