# Supplementary material for: Protein model accuracy estimation based on local structure quality assessment using 3D convolutional neural network
Source: PLoS One. 2019 Sep 5;14(9):e0221347. doi: 10.1371/journal.pone.0221347 (PMC6728020; doi:10.1371/journal.pone.0221347)
Supplement: S7 Table — The legend is the same as that for Table 4 for the first five columns. (DOCX) [file pone.0221347.s007.docx]

**S7 Table. Comparison with single-model methods in CASP11 stage2 without homologous proteins**

The legend is the same as that for Table 4 for the first five columns.

| Method | Pearson | Spearman | Loss | Rank |
| --- | --- | --- | --- | --- |
| Proposed | **0.483** | **0.448** | **5.092** | **27.099** |
| VoroMQA | 0.415 **(0.0027)** | 0.396 **(0.0214)** | 7.293 | 27.25 |
| MULTICOM-CLUSTER | 0.409 **(0.0009)** | 0.400 **(0.0274)** | 7.378 | 31.83 |
| MULTICOM-NOVEL | 0.392 **(0.0004)** | 0.390 **(0.0267)** | 7.185 | 32.375 |
| RFMQA | 0.385 **(2.95E-05)** | 0.368 **(0.0005)** | 7.253 | 31.621 |
| ProQ2 | 0.376 **(5.85E-06)** | 0.370 **(0.0008)** | 6.305 | 35.705 |
| ProQ2-refine | 0.374 **(4.08E-06)** | 0.380 **(0.0022)** | 6.689 | 34.67 |
