# Supplementary material for: Protein model accuracy estimation based on local structure quality assessment using 3D convolutional neural network
Source: PLoS One. 2019 Sep 5;14(9):e0221347. doi: 10.1371/journal.pone.0221347 (PMC6728020; doi:10.1371/journal.pone.0221347)
Supplement: S8 Table — The legend is the same as that for Table 4 for the first five columns. (DOCX) [file pone.0221347.s008.docx]

**S8 Table. Comparison with single-model methods in CASP12 stage1 without homologous proteins**

The legend is the same as that for Table 4 for the first five columns.

| Method | Pearson | Spearman | Loss | Rank |
| --- | --- | --- | --- | --- |
| Proposed | **0.697** | 0.480 | **2.161** | **2.231** |
| MULTICOM-CLUSTER | 0.673 (0.1270) | 0.601 **(0.0002)** | 7.076 | 2.614 |
| ProQ3 | 0.659 **(0.0126)** | 0.635 **(1.51E-05)** | 3.865 | 2.42 |
| SVMQA | 0.651 **(0.0062)** | **0.646** **(7.25E-08)** | 3.154 | 2.414 |
| ProQ2 | 0.635 **(0.0045)** | 0.642 **(1.10E-07)** | 7.471 | 2.771 |
| VoroMQA | 0.614 **(3.47E-05)** | 0.555 **(0.0047)** | 7.607 | 2.743 |
