# Supplementary material for: Protein model accuracy estimation based on local structure quality assessment using 3D convolutional neural network
Source: PLoS One. 2019 Sep 5;14(9):e0221347. doi: 10.1371/journal.pone.0221347 (PMC6728020; doi:10.1371/journal.pone.0221347)
Supplement: S9 Table — The legend is the same as that for Table 4 for the first five columns. (DOCX) [file pone.0221347.s009.docx]

**S9 Table. Comparison with single-model methods in CASP12 stage2 without homologous proteins**

The legend is the same as that for Table 4 for the first five columns.

| Method | Pearson | Spearman | Loss | Rank |
| --- | --- | --- | --- | --- |
| Proposed | **0.657** | 0.591 | 6.439 | **16.818** |
| ProQ3 | 0.636 **(0.0405)** | 0.593 (0.7913) | 5.893 | 20.343 |
| SVMQA | 0.626 (0.3866) | **0.594** (0.2737) | **5.475** | 20.743 |
| VoroMQA | 0.590 **(3.78E-05)** | 0.549 **(0.0134)** | 8.003 | 19.914 |
| ProQ2 | 0.588 **(0.0001)** | 0.559 **(0.0411)** | 6.902 | 20.843 |
| MULTICOM-CLUSTER | 0.568 **(2.07E-05)** | 0.541 **(0.0739)** | 7.966 | 24.543 |
