# Supplementary material for: Protein model accuracy estimation based on local structure quality assessment using 3D convolutional neural network
Source: PLoS One. 2019 Sep 5;14(9):e0221347. doi: 10.1371/journal.pone.0221347 (PMC6728020; doi:10.1371/journal.pone.0221347)
Supplement: S10 Table — The first column represents the method name. The second and third columns, respectively, represent the average Pearson’s correlation coefficient (Pearson) and average Spearman’s correlation (Spearman) between the actual ranking and predicted ranking. The fourth column represents the average TMscore loss. Native structures were removed. (DOCX) [file pone.0221347.s010.docx]

**S10 Table. Comparison with single-model methods in I-TASSER**

The first column represents the method name. The second and third columns, respectively, represent the average Pearson’s correlation coefficient (Pearson) and average Spearman’s correlation (Spearman) between the actual ranking and predicted ranking. The fourth column represents the average TMscore loss. Native structures were removed.

| Method | Pearson | Spearman | TMscore loss |
| --- | --- | --- | --- |
| Proposed | 0.491 | 0.433 | 0.103 |
| SVMQA | **0.551** | **0.458** | **0.088** |
| RWplus | 0.488 | 0.416 | 0.101 |
| GOAP | 0.477 | 0.392 | 0.111 |
| OPUS-PSP | 0.282 | 0.286 | 0.130 |
