# Supplementary material for: Protein model accuracy estimation based on local structure quality assessment using 3D convolutional neural network
Source: PLoS One. 2019 Sep 5;14(9):e0221347. doi: 10.1371/journal.pone.0221347 (PMC6728020; doi:10.1371/journal.pone.0221347)
Supplement: S11 Table — The legend is the same as that for S11 Table for the first four columns. (DOCX) [file pone.0221347.s011.docx]

**S11 Table. Comparison with single-model methods in 3DRobot**

The legend is the same as that for Table S11 for the first four columns.

| Method | Pearson | Spearman | TMscore loss |
| --- | --- | --- | --- |
| Proposed | **0.928** | 0.878 | **0.013** |
| SVMQA | 0.910 | **0.882** | 0.035 |
| OPUS-PSP | 0.807 | 0.752 | 0.036 |
| GOAP | 0.883 | 0.849 | 0.052 |
| RWplus | 0.834 | 0.806 | 0.071 |
