# Supplementary material for: Protein model accuracy estimation based on local structure quality assessment using 3D convolutional neural network
Source: PLoS One. 2019 Sep 5;14(9):e0221347. doi: 10.1371/journal.pone.0221347 (PMC6728020; doi:10.1371/journal.pone.0221347)
Supplement: S12 Table — First column represents method name. Second and third columns represent AUC and Pearson value of local assessment. (DOCX) [file pone.0221347.s012.docx]

**S12 Table. Local assessment performance comparison on CASP12 stage2 dataset.**

First column represents method name. Second and third columns represent AUC and Pearson value of local assessment.

| Method | AUC | Pearson |
| --- | --- | --- |
| Proposed | 0.846 | 0.355 |
| ProQ3 | **0.878** | **0.507** |
| ProQ2 | 0.842 | 0.462 |
| VoroMQA | 0.808 | 0.368 |
