# Supplementary material for: Protein model accuracy estimation based on local structure quality assessment using 3D convolutional neural network
Source: PLoS One. 2019 Sep 5;14(9):e0221347. doi: 10.1371/journal.pone.0221347 (PMC6728020; doi:10.1371/journal.pone.0221347)
Supplement: S13 Table — The legend is the same as that for Table 3 for the first six columns. (DOCX) [file pone.0221347.s013.docx]

**S13 Table. Comparison with the method using only core residues local assessment in CASP11 stage2**

The legend is the same as that for Table 3 for the first six columns.

| Dataset | Method | Pearson | Spearman | Loss | Rank |
| --- | --- | --- | --- | --- | --- |
| CASP11 stage2 | Proposed | **0.495** | **0.465** | **4.580** | **23.400** |
|  | Core residue | 0.469 | 0.440 | 4.938 | 26.325 |
