# Supplementary material for: Protein model accuracy estimation based on local structure quality assessment using 3D convolutional neural network
Source: PLoS One. 2019 Sep 5;14(9):e0221347. doi: 10.1371/journal.pone.0221347 (PMC6728020; doi:10.1371/journal.pone.0221347)
Supplement: S1 Fig — The neural network architecture is shown. (DOCX) [file pone.0221347.s014.docx]

**S1 Fig. Convolutional neural network architecture**

The neural network architecture is shown.
