# Supplementary material for: Protein model accuracy estimation based on local structure quality assessment using 3D convolutional neural network
Source: PLoS One. 2019 Sep 5;14(9):e0221347. doi: 10.1371/journal.pone.0221347 (PMC6728020; doi:10.1371/journal.pone.0221347)
Supplement: S3 Fig — Loss values and validation MCC are shown. (DOCX) [file pone.0221347.s016.docx]

S3 Fig. Loss and MCC during training

Loss values and validation MCC are shown.
